# Risk factors for misclassification in predicting EGFR mutation status using PET/CT imaging in non-small cell lung cancer patients

**DOI:** 10.3389/fonc.2025.1702905

**Published:** 2025-12-03

**Authors:** Jiali Li, Zihang Zeng, Jie Chen, Tianxing Fang, Hongjun Liu, Yong He

**Affiliations:** 1 Department of Nuclear Medicine, Zhongnan Hospital of Wuhan University, Wuhan, China; 2 Department of Radiation and Medical Oncology, Zhongnan Hospital of Wuhan University, Wuhan, China

**Keywords:** EGFR mutation, misclassification, non-small cell lung cancer, ^18^F-FDG PET/CT, radiomics

## Abstract

**Objective:**

This study aims to develop 10 machine learning models based on positron emission tomography/computed tomography (PET/CT) radiomic features to predict epidermal growth factor receptor (EGFR) mutations in non-small cell lung cancer (NSCLC) patients and to identify risk factors contributing to model misclassification.

**Methods:**

This study included 277 NSCLC patients from Zhongnan Hospital, Wuhan University, who underwent pretreatment ^18^F-FDG PET/CT and EGFR mutation testing. A PET/CT signature (PCS)-nomogram was developed by comparing 10 machine learning algorithms for EGFR prediction. Leave-one-out cross-validation generated model-specific EGFR mutation probabilities for individual patients, and performance disparities were analyzed across clinical subgroups. Model performance was assessed using the receiver operating characteristic curve, Youden’s index, decision curve analysis, and DeLong’s test.

**Results:**

The PCS-nomogram model, constructed using the partial least squares generalized linear models (plsRglm) algorithm, achieved optimal performance in predicting EGFR mutations in NSCLC patients (training cohort: area under the curve [AUC] = 0.80; validation cohort: AUC = 0.82). Smoking history caused statistically significant performance deterioration in seven of 10 machine learning models (|ΔYouden’s index| ≥ 0.1). The PCS model demonstrated higher predictive performance in never-smokers than in smokers (AUC = 0.90 vs. 0.64; *p* < 0.05).

**Conclusion:**

A plsRglm-based PCS-nomogram model was proposed for the noninvasive prediction of EGFR mutations in NSCLC patients. Compared with smokers, radiomics-based EGFR mutation prediction demonstrated superior performance in never-smokers.

## Introduction

Lung cancer remains the leading cause of cancer-related morbidity and mortality worldwide, with non-small cell lung cancer (NSCLC) accounting for approximately 85% of all cases (1, 2). The advent of targeted therapies has substantially improved the prognosis of patients harboring epidermal growth factor receptor (EGFR) mutations, with EGFR tyrosine kinase inhibitors (TKIs) providing markedly longer progression-free survival compared with chemotherapy (18.9 months vs. 6.3 months) in NSCLC patients (3, 4). Currently, EGFR mutation testing relies on tissue biopsy specimens. However, this approach is invasive, costly, and often limited by tissue availability, posing challenges for routine genetic testing. These limitations highlight the need for reliable, noninvasive methods to determine EGFR mutation status.

^18^F-fluorodeoxyglucose (^18^F-FDG) positron emission tomography/computed tomography (PET/CT) is a widely used imaging modality for the diagnosis and staging of NSCLC (5, 6). By combining anatomical and metabolic information, PET/CT provides unique insights into tumor biology (7). With advancements in radiomics and machine learning algorithms, researchers have introduced a new paradigm of “from imaging to molecular diagnosis” (8, 9).

Recent studies have explored radiomics models derived from PET, CT, or integrated PET/CT data to predict EGFR mutation status; however, their predictive performance has been inconsistent, with reported areas under the curve (AUCs) ranging from 0.58 to 0.94 (10–12). This instability poses significant challenges to clinical application and may be attributed to heterogeneity in patient populations and imaging protocols (13, 14). Such variability also arises from differences in feature selection strategies. Although these studies help explain heterogeneity in overall model performance, the specific clinical and tumor-related factors contributing to model misclassification at the individual patient level remain largely unexplored. Identifying these determinants is crucial for understanding sources of predictive variability and for delineating patient subpopulations most suitable for radiomics-based EGFR prediction.

In this study, we developed a PET/CT signature (PCS)-nomogram model using 10 machine learning approaches to noninvasively predict *EGFR* mutation status in NSCLC patients. Crucially, we investigated the essential clinical determinants of predictive failures and identified a specific patient subpopulation with enhanced suitability for radiomics-based EGFR prediction.

## Materials and methods

### Patient selection

**Figure 1:**
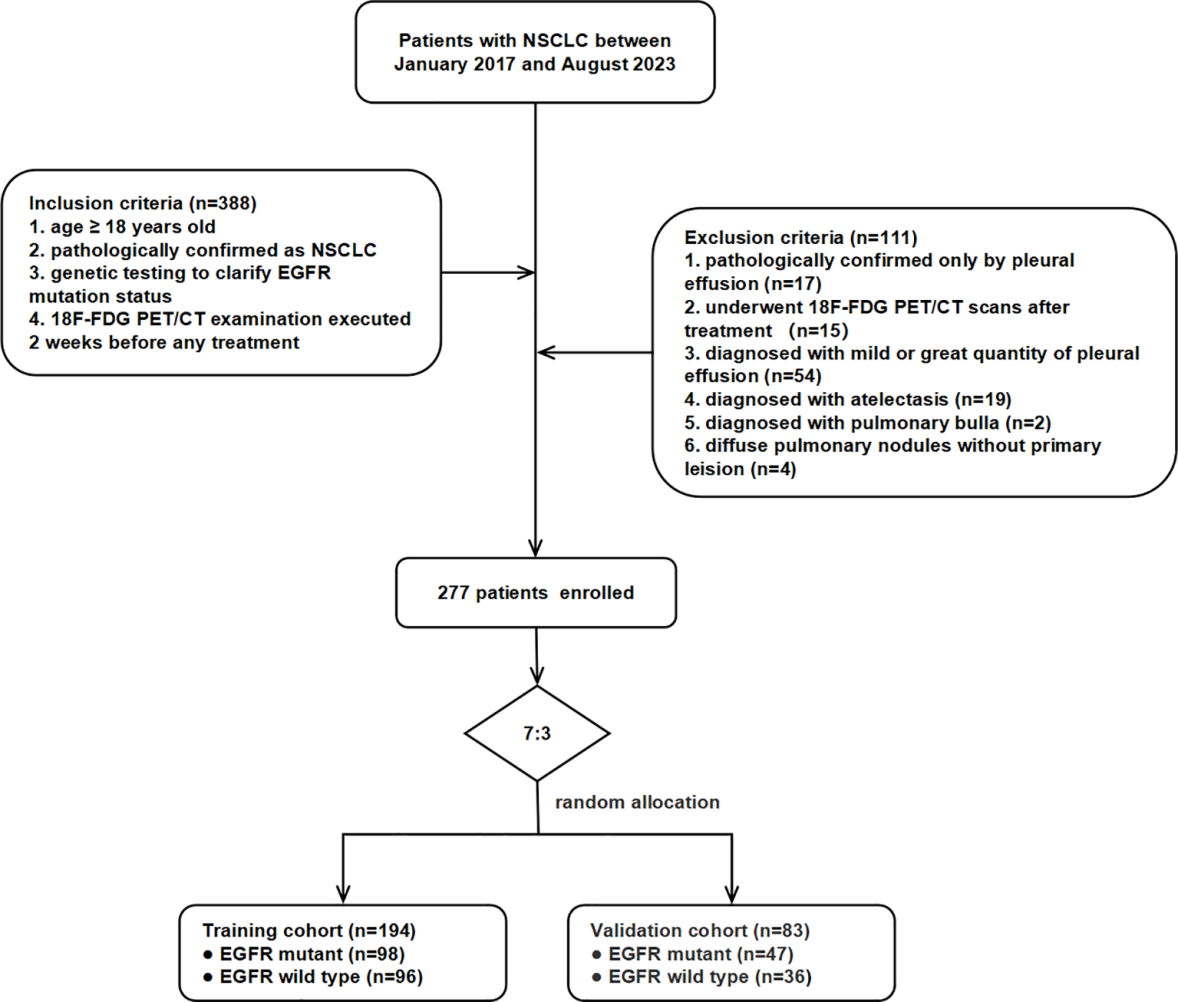
Patient inclusion and exclusion flowchart.

This retrospective study was approved by the Institutional Review Board of Zhongnan Hospital of Wuhan University and conducted in accordance with the Declaration of Helsinki. Informed consent was waived due to the study’s retrospective nature. This study included patients pathologically diagnosed with NSCLC who underwent EGFR mutation testing and ^18^F-FDG PET/CT prior to therapy at Zhongnan Hospital of Wuhan University between January 2017 and August 2023. Initially, 388 patients were collected, of whom 277 met the inclusion criteria and were enrolled. Inclusion criteria were as follows (1): age ≥ 18 years old (2); histologically confirmed NSCLC via surgery or biopsy (3); genetic testing for *EGFR* mutation status; and (4) ^18^F-FDG PET/CT examination performed within 2 weeks prior to initial treatment. Exclusion criteria were as follows (1): pathological confirmation only by pleural effusion (17 cases) (2); ^18^F-FDG PET/CT scans performed after therapy (15 cases) (3); mild or extensive pleural effusion (54 cases) (4); segmental or extensive atelectasis (19 cases) (5); bullous emphysema (two cases); and (6) diffuse pulmonary nodules without a dominant primary lesion (four cases). The 277 participants were randomly divided into training (*n* = 194) and validation (*n* = 83) cohorts at a ratio of 7:3. The workflow of patient selection is shown in **Figure 1**.

### EGFR mutation detection

Histological specimens were obtained via surgery, biopsy, or endobronchial ultrasound-guided transbronchial needle aspiration puncture. *EGFR* mutation status was determined using amplification refractory mutation system polymerase chain reaction or high-throughput sequencing. EGFR mutations encompass a wide spectrum of subtypes, including exon 19 deletions, exon 21 L858R, exon 20 insertions, exon 18 G719X, and various compound mutations. Since other *EGFR* mutation subtypes had relatively few positive cases, except for exon 19 deletions and exon 21 L858R, all samples harboring missense mutations, insertions, or deletions on exons 18–21 were classified as *EGFR* mutant to minimize class imbalance introduced by excessive subdivision. The remaining samples were classified as *EGFR* wild-type.

### PET/CT image acquisition

All participants underwent ^18^F-FDG PET/CT examination following a standardized imaging protocol. ^18^F-FDG (radiochemical purity of > 95%) was purchased from HTA Co., Ltd. (Wuhan, China). Participants fasted for at least 6 h to ensure blood glucose levels < 11.0 mmol/L and were intravenously injected with ^18^F-FDG at 3.70 MBq/kg. Imaging was performed using a Siemens Biograph mCT PET/CT scanner (Siemens Healthineers, Erlangen, Germany) after a 60 min ± 5 min uptake period at rest. Low-dose CT scans were acquired with a 2-mm slice thickness (matrix 512 × 512) for attenuation correction. PET images were acquired in three-dimensional (3D) mode with six to eight bed positions (2.5 min per bed, 2 mm thickness, matrix 200 × 200) and reconstructed using the TrueX and ultra-high-definition PET algorithms embedded in the MMWP workstation.

### Image segmentation and feature extraction

Image segmentation and feature extraction were performed using the PyRadiomics package (15) implemented in 3D Slicer software (version 5.5.0) by two radiologists with over 3 years of experience. Prior to segmentation, PET and CT images were registered using the SlicerElastix module with the “generic rigid” method. Semiautomatic contouring of the primary tumor volume of interest (VOI) was then conducted on PET/CT fusion images. CT images were displayed with a lung window (WL = − 600 HU, WW = 1,500 HU), and regions with standardized uptake values (SUV) ≥ 40% of SUVmax were defined as VOIs. Manual adjustment was applied when the lesion was adjacent to the mediastinum or chest wall.

Prior to feature extraction, both PET and CT images and their corresponding masks were resampled to an isotropic voxel size of 2.0 mm^3^ × 2.0 mm^3^ × 2.0 mm^3^ using B-spline interpolation for images and nearest-neighbor interpolation for masks to ensure spatial consistency. Wavelet decomposition was applied to the resampled images to enhance multiscale textural representation. Radiomic features were extracted separately from PET (bin width = 0.4) and CT (bin width = 25) images. Extracted features included shape (2D and 3D), first-order statistics, and texture features based on the Gray Level Co-occurrence Matrix (GLCM), Gray Level Dependence Matrix (GLDM), Gray Level Run Length Matrix (GLRLM), Gray Level Size Zone Matrix (GLSZM), and Neighboring Gray Tone Difference Matrix (NGTDM).

### Radiomics feature selection and PCS development

Radiomics feature selection was performed using a four-step approach. First, intraclass correlation coefficients (ICCs) were calculated to assess interobserver reproducibility, and features with ICC > 0.8 were retained. Subsequent steps were conducted in the training cohort (*n* = 194). Second, univariate analysis was applied using Student’s *t*-tests or Mann–Whitney *U* tests, depending on normality; features with an adjusted *p* < 0.20 were retained and standardized by *Z*-score transformation. Third, the least absolute shrinkage and selection operator (LASSO) logistic regression was used to identify predictive features with nonzero coefficients, with AUC serving as the evaluation metric during cross-validation. Finally, features with high correlation (Pearson’s *r* > 0.6) were removed to minimize redundancy.

The selected radiomics features were then used to develop predictive models with 10 machine learning algorithms in the training cohort: partial least squares generalized linear models (plsRglm), NaiveBayes, Ridge, gradient boost machine (GBM), support vector machine (SVM), linear discriminant analysis (LDA), LASSO (16), elastic-net (Enet), generalized linear models with boosting (glmBoost), and extreme gradient boosting (XGBoost) (16–20). Model performance was evaluated using AUC, accuracy, sensitivity, specificity, negative predictive value, and positive predictive value. AUCs were compared using the DeLong test.

### Clinical feature selection

Clinical features were screened through a four-step process within the training cohort. Before feature selection, categorical clinical features were preprocessed. Binary categorical features (e.g., gender, smoking history) were encoded as 0/1. Ordinal features, including the American Joint Committee on Cancer (AJCC) stage, were encoded as ordered integers reflecting disease severity. The feature selection pipeline was then conducted. First, univariate analyses (Student’s *t*-test or Mann–Whitney *U* test for continuous features; Chi-square test for categorical features) were conducted to identify candidates with *p* < 0.05. Second, LASSO logistic regression was performed to identify features with nonzero coefficients. Third, interfeature correlations were examined using Pearson’s correlation (continuous) or Spearman’s correlation (categorical), and features with |*r*| ≥ 0.6 were removed. Finally, features with both *p* < 0.05 in univariate logistic regression and *p* < 0.20 in multivariable logistic regression were retained for nomogram construction.

### Construction and evaluation of PCS-nomogram

A PCS-nomogram was developed by integrating the PCS and selected clinical features using logistic regression to predict EGFR mutation status. Calibration curves were plotted to assess the agreement between predicted and observed mutation status, while decision curve analysis (DCA) was performed to evaluate clinical benefits across different threshold probabilities.

### Leave-one-out cross-validation

Leave-one-out cross-validation (LOOCV), a type of cross-validation, was applied to evaluate the model performance, in which each sample was used once as a validation case while the remaining samples served as the training set. To identify clinical risk factors for prediction failure, LOOCV was performed across the entire cohort (*n* = 277) to calculate the accuracy of the radiomics-based model for each of the 10 machine-learning algorithms.

### Statistical analysis

All statistical analyses were performed using R software (version 4.3.3). Continuous variables were compared using Student’s *t*-test or the Mann–Whitney *U* test, while categorical variables were analyzed with the Chi-square test. Multiple testing was corrected using the Benjamini–Hochberg method. Machine learning models were implemented using the R packages “plsRglm”, “Glmnet”, “gbm”, “MASS”, “mboost”, and “xgboost”. ROC curves, calibration curves, and DCA plots were generated with the “pROC”, “rms”, and “rmda” packages, respectively. Triangular correlation plots were created using “linkET”, and other plots were generated with “ggplot”. A two-sided *p* < 0.05 was considered statistically significant.

## Results

### Patient characteristics

A total of 277 patients meeting the inclusion criteria were randomly assigned to the training (*n* = 194/277, 70%; mean age, 63.33 ± 10.22) and validation cohorts (*n* = 83/277, 30%; mean age, 62.41 ± 10.27). No significant differences were observed between the cohorts in terms of age, gender, smoking history, AJCC stage, and pathological type (all *p >*0.05). The prevalence of *EGFR* mutation was balanced, with 98 (50.52%) and 47 (56.62%) positive cases in the training and validation cohorts, respectively (*p* > 0.05). Patient clinical characteristics are summarized in **Table 1**.

**Table 1:** Clinical characteristics of lung cancer patients in the training and validation cohorts.

Characteristics	Training cohort (N = 194)	Validation cohort (N = 83)	P-value
	No. (%)	No. (%)	
Gender			0.084
Female	79 (40.72%)	24 (28.92%)	
Male	115 (59.28%)	59 (71.08%)	
Age			0.495
Mean±SD	63.33±10.22	62.41±10.27	
BMI			0.884
Mean±SD	23.01±2.90	22.95±3.00	
Smoking History			0.159
No	117 (61.26%)	42 (51.22%)	
Yes	74 (38.74%)	40 (48.78%)	
Alcohol history			0.473
No	22 (81.48%)	12 (70.59%)	
Yes	5 (18.52%)	5 (29.41%)	
CEA			0.602
Median(IQR)	7.70 (3.08-38.10)	6.70(2.64-68.13)	
SCC.Ag			0.328
Median(IQR)	0.80(0.51-1.21)	0.82(0.67-1.08)	
CA125			0.590
Median(IQR)	22.72(11.28-78.04)	25.74(15.04-77.23)	
NSE			0.336
Median(IQR)	13.79(11.41-17.02)	13.10(11.83-18.75)	
CA211			0.550
Median(IQR)	5.20(3.06-13.50)	4.16(2.74-11.16)	
T			0.397
1	45 (23.44%)	19 (22.89%)	
2	51 (26.56%)	20 (24.1%)	
3	28 (14.58%)	19 (22.89%)	
4	68 (35.42%)	25 (30.12%)	
N			0.044
0	50 (26.32%)	15 (18.52%)	
1	17 (8.95%)	2 (2.47%)	
2	52 (27.37%)	33 (40.74%)	
3	71 (37.37%)	31 (38.27%)	
M			0.554
0	76 (39.58%)	29 (34.94%)	
1	116 (60.42%)	54 (65.06%)	
AJCC stage			0.555
I	25 (13.16%)	6 (7.23%)	
II	12 (6.32%)	5 (6.02%)	
III	37 (19.47%)	18 (21.69%)	
IV	116 (61.05%)	54 (65.06%)	
Pathological type			0.101
Squamous cell carcinoma	17 (8.76%)	5 (6.02%)	
Adenocarcinoma	169 (87.11%)	70 (84.34%)	
Adenosquamous carcinoma	2 (1.03%)	1 (1.2%)	
Other	6 (3.09%)	7 (8.43%)	
Ki.67			0.704
Mean±SD	0.31±0.27	0.32±0.26	
EGFR status			0.352
wild type	96 (49.48%)	36 (43.37%)	
EGFR mutant	98 (50.52%)	47 (56.63%)	

### PET/CT feature extraction and selection

**Figure 2:**
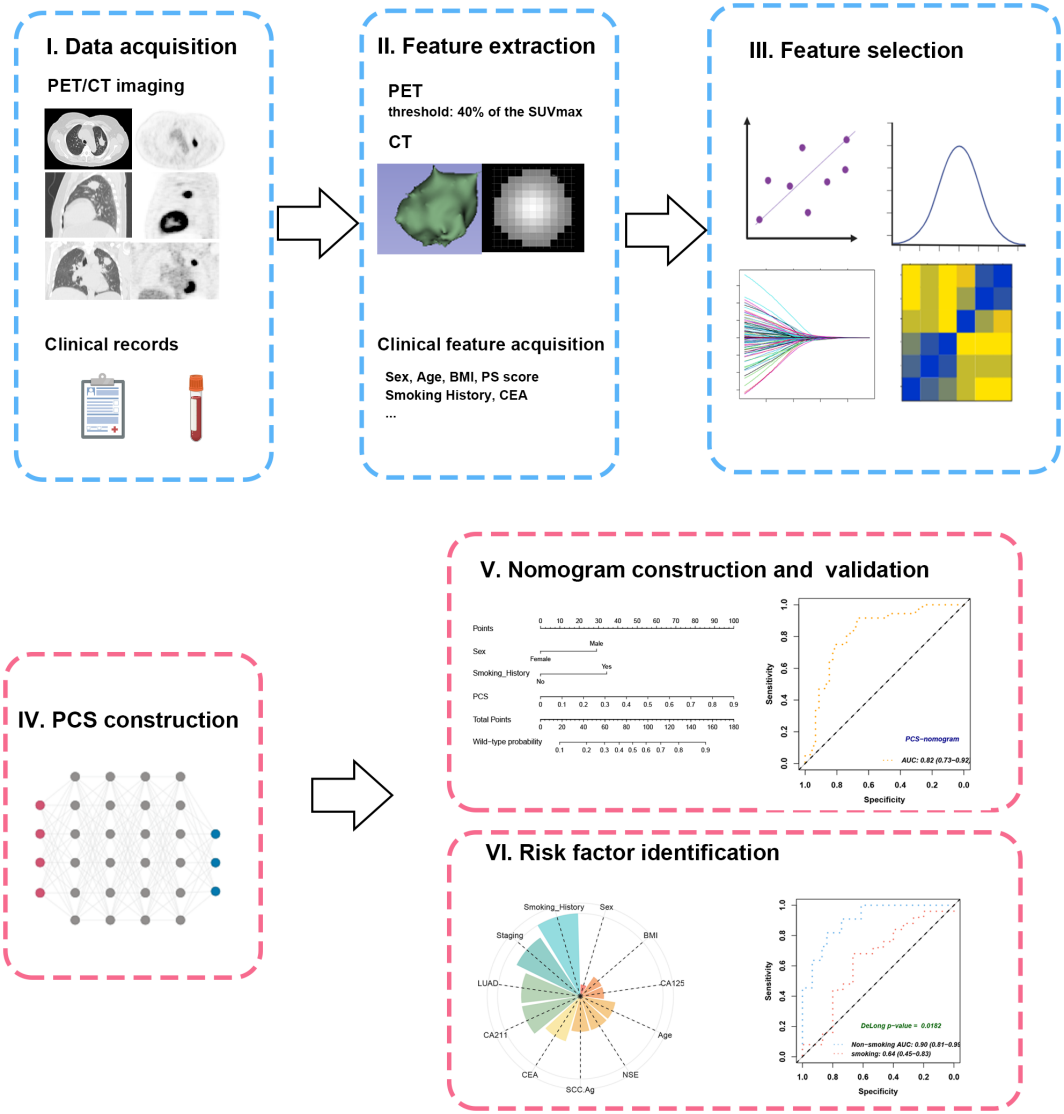
Workflow of study design. (I) The PET/CT imaging and clinical records of the included patients were collected and subjected to feature extraction. (II) The VOIs were semiautomatically contoured at the threshold of 40% of SUVmax. (III) Feature selection was enforced through ICC, U-test, LASSO, and Pearson’s correlation. (IV) The PCS was developed from combined radiomics by plsRglm. (V) The PCS was further integrated with significant clinical features to create a PCS-nomogram. (VI) Subgroup analyses revealed the impacts of different clinical characteristics on PCS. VOI, volume of interest; SUVmax, maximal standard uptake value; ICC, intraclass correlation coefficient; PCS, PET/CT signature.

**Figure 3:**
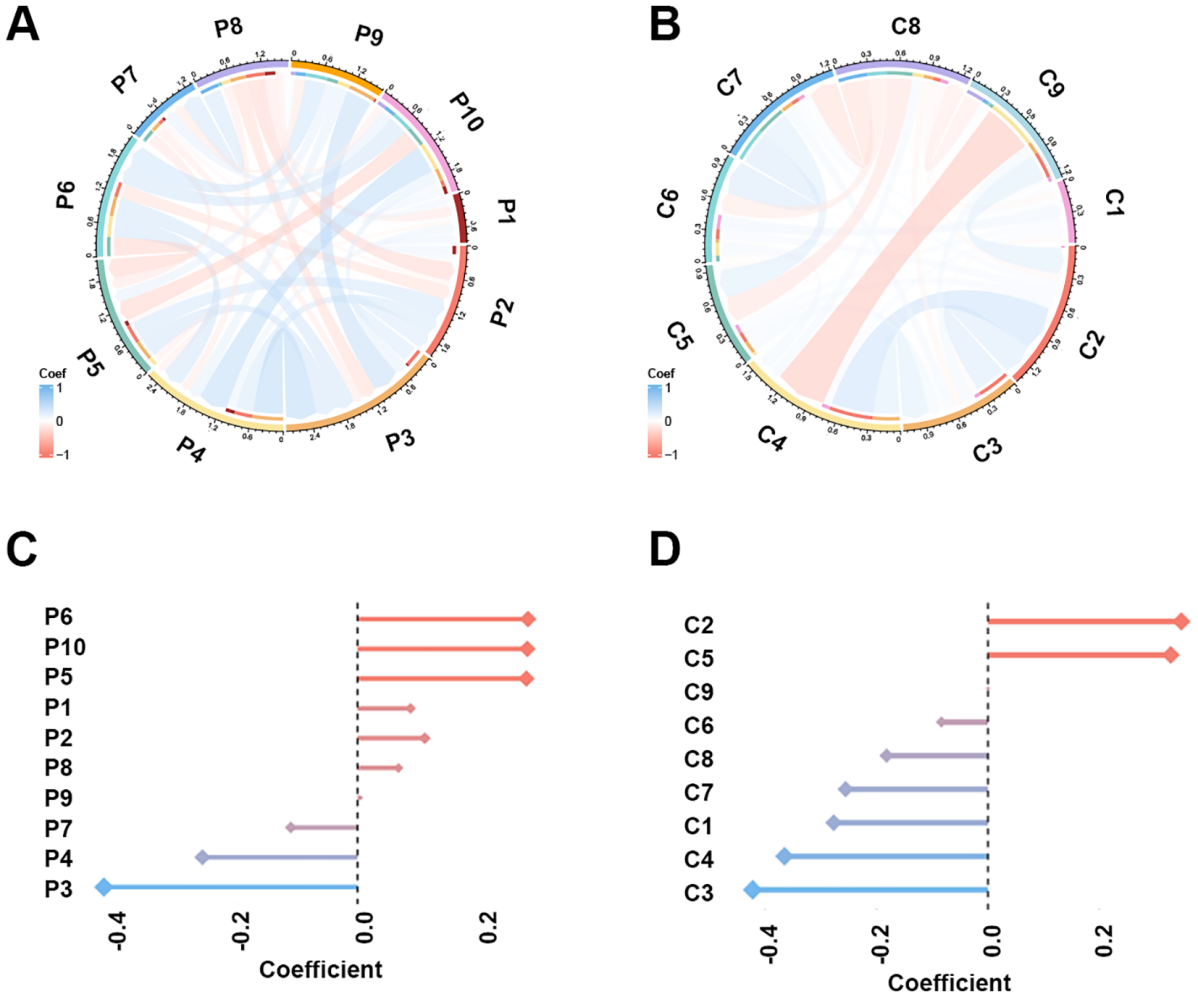
The Pearson’s correlation and LASSO coefficient weight of the selected 10 PET features and nine CT features. **(A)** Chord plot of the correlation between PET features. Color represents Pearson’s correlation coefficient. **(B)** Chord plot of the correlation between CT features. **(C)** LASSO coefficient weight of each PET feature. The dependent variable in the LASSO regression was EGFR mutation. **(D)** LASSO coefficient weight of each CT feature.

The study workflow is illustrated in **Figure 2**. A total of 851 PET features and 851 CT features were independently extracted from the images by two experienced radiologists. Feature reproducibility was assessed using the intraclass correlation coefficient (ICC), and 566 PET (66.5%) and 580 CT (68.2%) features with excellent reliability (ICC > 0.8) were retained for further analysis. Univariate analysis using Student’s *t*-test or Mann–Whitney *U* test identified 158 PET and 16 CT features with *p* < 0.2, which were then subjected to LASSO regression to select the optimal feature combination for model construction (**Supplementary Figure S1**). Highly correlated features (Pearson’s correlation coefficient > 0.6) were excluded to reduce redundancy. Ultimately, 10 PET and nine CT features were selected (**Table 2**). The chord diagram demonstrated the absence of significant collinearity among the selected PET (**Figure 3A**) and CT (**Figure 3B**) radiomic features. Notably, PET features P3 (original_glszm_ZoneEntropy) and P6 (wavelet-LHL_gldm_SmallDependenceEmphasis), as well as CT features C3 (wavelet-LLH_glszm_SmallAreaEmphasis) and C4 (wavelet-LHH_gldm_DependenceEntropy), exhibited the highest coefficient weights related to *EGFR* mutation (**Figures 3C, D**, **Table 2**).

**Table 2:** The coefficient weights of the selected features used for the radiomics-based model in LASSO regression.

Source	Radiomic features	Abbreviation	Coefficient weight
PET	original_firstorder_Kurtosis	P1	0.11
PET	original_glcm_Imc1	P2	0.09
PET	original_glszm_ZoneEntropy	P3	− 0.42
PET	wavelet-LLH_firstorder_Energy	P4	− 0.26
PET	wavelet-LLH_glcm_InverseVariance	P5	0.28
PET	wavelet-LHL_gldm_SmallDependenceEmphasis	P6	0.28
PET	wavelet-LHH_glcm_DifferenceAverage	P7	− 0.11
PET	wavelet-LHH_gldm_DependenceNonUniformityNormalized	P8	0.07
PET	wavelet-HLL_glcm_MCC	P9	0.01
PET	wavelet-HLH_glcm_ClusterShade	P10	0.28
CT	original_shape_Sphericity	C1	− 0.28
CT	original_ngtdm_Complexity	C2	0.35
CT	wavelet-LLH_glszm_SmallAreaEmphasis	C3	− 0.42
CT	wavelet-LHH_gldm_DependenceEntropy	C4	− 0.37
CT	wavelet-HLH_glcm_ClusterShade	C5	0.33
CT	wavelet-HHL_firstorder_Mean	C6	− 0.08
CT	wavelet-HHL_glcm_ClusterShade	C7	− 0.26
CT	wavelet-HHH_glcm_ClusterShade	C8	− 0.18
CT	wavelet-HHH_gldm_DependenceNonUniformityNormalized	C9	0.00
Clinical	Gender	–	0.47
Clinical	Smoking_History	–	0.47
Clinical	Pathological_type	–	− 0.55

### Performance of 10 radiomics-based machine learning models in predicting EGFR mutations

**Figure 4:**
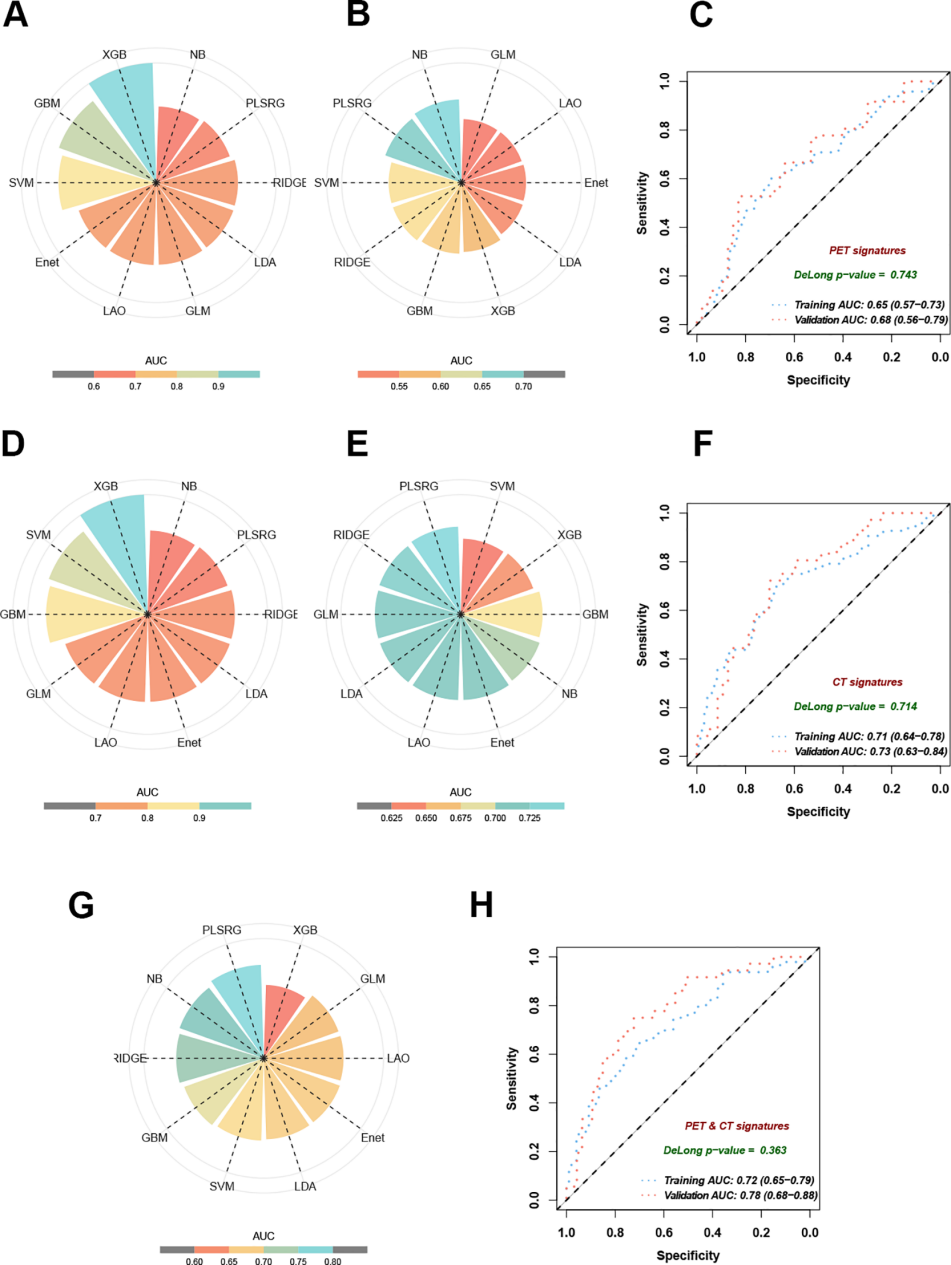
Performance of 10 machine learning algorithms on training and validation datasets. **(A)** AUC in training cohorts based on selected PET features. **(B)** AUC in validation cohorts based on selected PET features. **(C)** The ROC curves demonstrated the model performance established with the plsRglm algorithm in training and validation cohorts based on selected PET features. **(D)** AUC in training cohorts based on selected CT features. **(E)** AUC in validation cohorts based on selected CT features. **(F)** The ROC curves demonstrate the model performance established with the plsRglm algorithm in training and validation cohorts based on selected CT features. **(G)** AUC in validation cohorts based on the plsRglm-based PCS model. **(H)** The ROC curves demonstrated the PCS model’s performance in training and validation cohorts.

Ten state-of-the-art machine learning algorithms, including plsRglm, NaiveBayes, Ridge, GBM, SVM, LDA, LASSO, Enet, glmBoost, and XGBoost, were benchmarked to identify the optimal predictive model for EGFR mutation in NSCLC patients. Among these, the plsRglm algorithm achieved the best performance. Radar plots illustrated the AUCs of all models in the training and validation cohorts based on PET features (**Figures 4A, B**, **Supplementary Table S1**). The plsRglm-based PET model achieved an AUC of 0.68 (95% confidence interval [CI]: 0.56–0.79) in the validation cohort (**Figure 4C**). Similarly, models were constructed based on CT features (**Figures 4D, E**, **Supplementary Table S2**), with the plsRglm-based CT model achieving optimal classification performance (AUC = 0.73; 95% CI: 0.63–0.84; **Figure 4F**). Dual-modality PET/CT models (PCS models) integrating the selected PET and CT features were further developed (**Figure 4G**, **Table 3**). The plsRglm-based PCS model demonstrated the highest performance in the validation cohort (AUC = 0.78; 95% CI: 0.68–0.88; **Figure 4H**). Moreover, the integrated PET/CT model significantly outperformed the PET-only model (DeLong test, *p* < 0.05) and showed a trend toward better performance compared with the CT-only model (DeLong test, *p* = 0.093).

**Table 3:** The diagnostic performance of 10 different radiomics-based models in predicting EGFR mutation status in the training and validation cohorts.

Task	Model_name	AUC	Accuracy	Sensitivity	Specificity	PPV	NPV
Training	XGB	1 (1.00–1.00)	1 (1.00–1.00)	1 (0.96–1)	1 (0.96–1)	1 (0.98–1)	1 (0.96–1)
Training	GBM	0.93 (0.9–0.96)	0.86 (0.8–0.91)	0.81 (0.72–0.88)	0.94 (0.87–0.97)	0.95 (0.9–0.97)	0.77 (0.69–0.83)
Training	SVM	0.91 (0.87–0.95)	0.85 (0.79–0.9)	0.85 (0.77–0.91)	0.85 (0.77–0.91)	0.86 (0.8–0.9)	0.84 (0.76–0.9)
Training	LDA	0.78 (0.72–0.85)	0.73 (0.66–0.79)	0.78 (0.69–0.85)	0.69 (0.59–0.77)	0.64 (0.57–0.71)	0.81 (0.73–0.87)
Training	LAO	0.78 (0.72–0.85)	0.72 (0.65–0.78)	0.74 (0.65–0.82)	0.7 (0.6–0.78)	0.68 (0.61–0.74)	0.76 (0.68–0.83)
Training	GLM	0.78 (0.72–0.85)	0.73 (0.66–0.79)	0.77 (0.68–0.84)	0.7 (0.6–0.78)	0.66 (0.59–0.73)	0.8 (0.72–0.86)
Training	Enet	0.78 (0.72–0.85)	0.72 (0.65–0.78)	0.77 (0.68–0.84)	0.68 (0.58–0.76)	0.63 (0.56–0.7)	0.8 (0.72–0.86)
Training	RIDGE	0.76 (0.7–0.83)	0.71 (0.64–0.77)	0.71 (0.61–0.79)	0.71 (0.61–0.79)	0.72 (0.65–0.78)	0.7 (0.61–0.77)
Training	PLSRG	0.72 (0.65–0.79)	0.68 (0.61–0.75)	0.67 (0.57–0.76)	0.69 (0.59–0.77)	0.71 (0.64–0.77)	0.65 (0.56–0.73)
Training	NB	0.72 (0.65–0.79)	0.68 (0.61–0.75)	0.67 (0.57–0.76)	0.69 (0.59–0.77)	0.71 (0.64–0.77)	0.65 (0.56–0.73)
Validation	PLSRG	0.78 (0.68–0.88)	0.73 (0.63–0.83)	0.79 (0.65–0.88)	0.68 (0.52–0.81)	0.72 (0.61–0.81)	0.75 (0.6–0.86)
Validation	NB	0.75 (0.64–0.85)	0.69 (0.58–0.78)	0.8 (0.67–0.89)	0.6 (0.44–0.74)	0.6 (0.49–0.7)	0.81 (0.66–0.9)
Validation	RIDGE	0.73 (0.62–0.84)	0.66 (0.55–0.76)	0.81 (0.68–0.9)	0.58 (0.42–0.73)	0.53 (0.42–0.63)	0.83 (0.68–0.92)
Validation	GBM	0.7 (0.59–0.82)	0.65 (0.54–0.75)	0.88 (0.76–0.95)	0.56 (0.4–0.71)	0.45 (0.35–0.55)	0.92 (0.77–0.98)
Validation	SVM	0.69 (0.57–0.8)	0.7 (0.59–0.79)	0.68 (0.54–0.8)	0.74 (0.58–0.86)	0.87 (0.77–0.93)	0.47 (0.34–0.6)
Validation	LDA	0.68 (0.56–0.79)	0.64 (0.53–0.74)	0.74 (0.6–0.84)	0.56 (0.4–0.71)	0.55 (0.44–0.66)	0.75 (0.6–0.86)
Validation	LAO	0.67 (0.55–0.78)	0.66 (0.55–0.76)	0.67 (0.53–0.79)	0.64 (0.48–0.78)	0.79 (0.68–0.87)	0.5 (0.37–0.63)
Validation	Enet	0.67 (0.56–0.79)	0.61 (0.5–0.72)	0.83 (0.7–0.91)	0.53 (0.37–0.68)	0.4 (0.3–0.51)	0.89 (0.74–0.96)
Validation	GLM	0.66 (0.55–0.78)	0.59 (0.48–0.7)	0.88 (0.76–0.95)	0.52 (0.36–0.67)	0.32 (0.23–0.42)	0.94 (0.79–0.98)
Validation	XGB	0.61 (0.49–0.73)	0.59 (0.48–0.7)	0.76 (0.62–0.86)	0.52 (0.36–0.67)	0.4 (0.3–0.51)	0.83 (0.68–0.92)

AUC, area under the curve; CI, confidence interval; PPV, positive predictive value; NPV, negative predictive value.

### Establishment and validation of the PCS-nomogram

**Figure 5:**
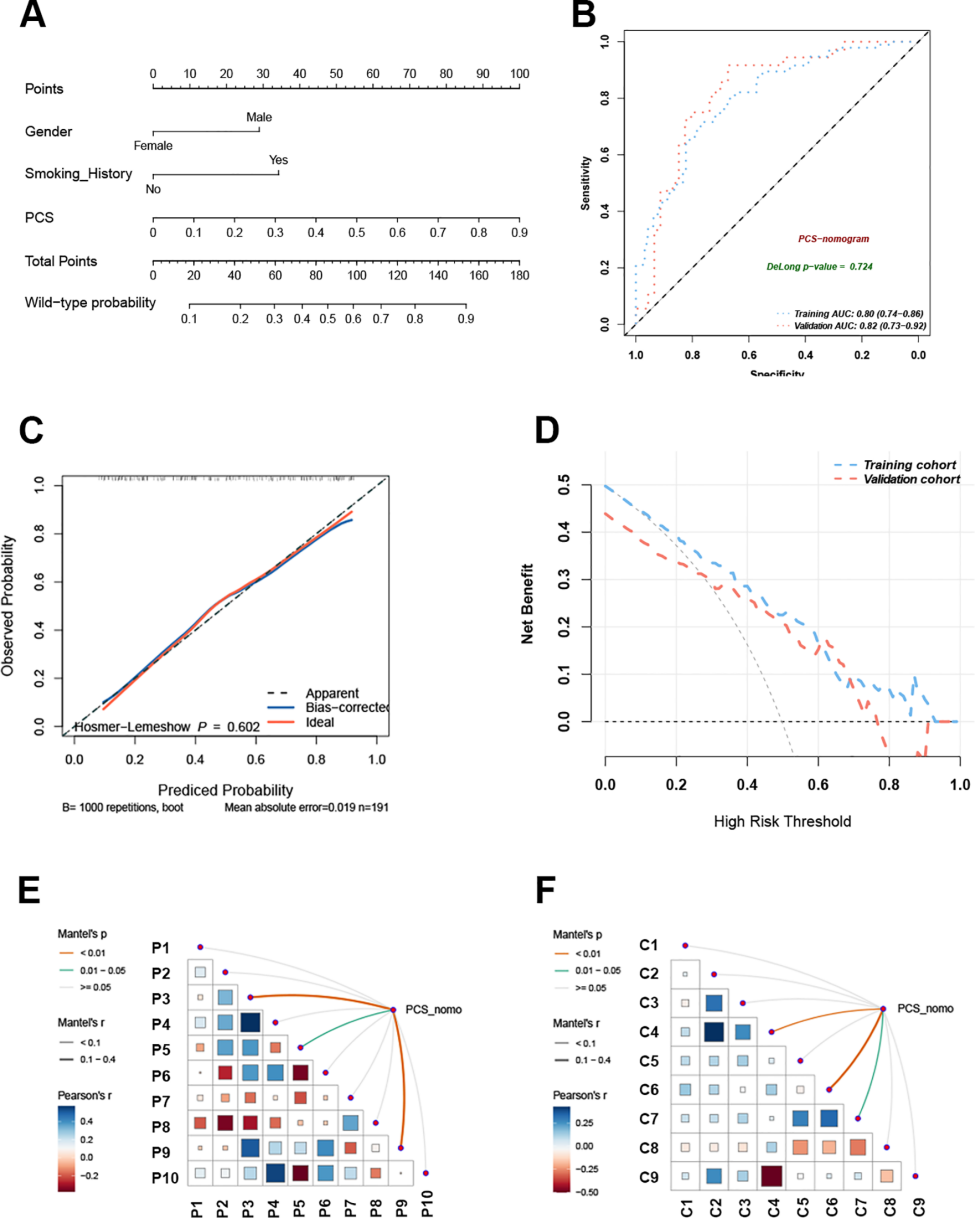
The PCS-nomogram model demonstrated outstanding capability in predicting EGFR mutations. **(A)** The nomogram model incorporates clinical features and PCS scores for predicting EGFR mutations in NSCLC patients. **(B)** The ROC curves of the PCS-nomogram model in the training and validation cohorts. **(C)** The calibration curve of the PCS-nomogram. **(D)** The DCA curves depict the net benefit at each decision threshold probability. **(E)** The triangular plot exhibits the correlation between PET features. **(F)** The triangular plot exhibits the correlation between CT features. PCS, PET/CT signature; ROC, receiver operating characteristic; DCA, decision curve analysis.

Clinical features are closely associated with patients’ *EGFR* mutation status (21). To improve predictive accuracy, key clinical features were incorporated into the PCS model. Gender, smoking history, and pathological type were identified through LASSO regression (**Table 2**), with gender and smoking history further confirmed as key predictors of EGFR mutation in univariate and multivariable logistic regression analyses (**Table 4**). A PCS-nomogram integrating the PCS and these clinical features was developed (**Figure 5A**). In the validation cohort, the model achieved an AUC of 0.82 (95% CI: 0.73–0.92; **Figure 5B**). Calibration plots demonstrated good agreement between predicted and observed EGFR mutation probabilities (**Figure 5C**), and decision curve analysis indicated a clinically meaningful net benefit (**Figure 5D**). Triangular correlation plots revealed significant associations between the PCS-nomogram and key radiomic features (*R* > 0.1, *p* < 0.05; **Figures 5E, F**).

**Table 4:** Univariate and multivariable logistic regression of PSC-nomogram.

Characteristics	Training cohort				Validation cohort			
	Univariate analysis	P-value	Multivariable analysis	P-value	Univariate analysis	P-value	Multivariable analysis	P-value
	OR (95% CI)		OR (95% CI)		OR (95% CI)		OR (95% CI)	
Gender	6.40 (3.41–12.45)	< 0.001	2.21 (0.98–5.02)	0.057	8.88 (2.70–40.58)	< 0.01	3.14 (0.73–16.67)	0.141
PCS	91.38 (17.54–549.11)	< 0.001	28.05 (4.33–201.93)	< 0.001	392.43 (23.73–10033.7)	< 0.001	114.32 (5.05–3735.13)	< 0.01
Smoking_History	6.22 (3.28–12.24)	< 0.001	2.89 (1.27–6.70)	< 0.05	4.70 (1.88–12.42)	< 0.01	2.60 (0.84–8.35)	0.099
Pathological_type	0.066 (0.01–0.23)	< 0.001	0.13 (0.019–0.51)	< 0.05	0.42 (0.12–1.37)	0.158	0.67 (0.15–2.80)	0.586

OR, odds ratio; CI, confidence interval; PSC, PET/CT signature.

### Identification of risk factors for prediction failure

**Figure 6:**
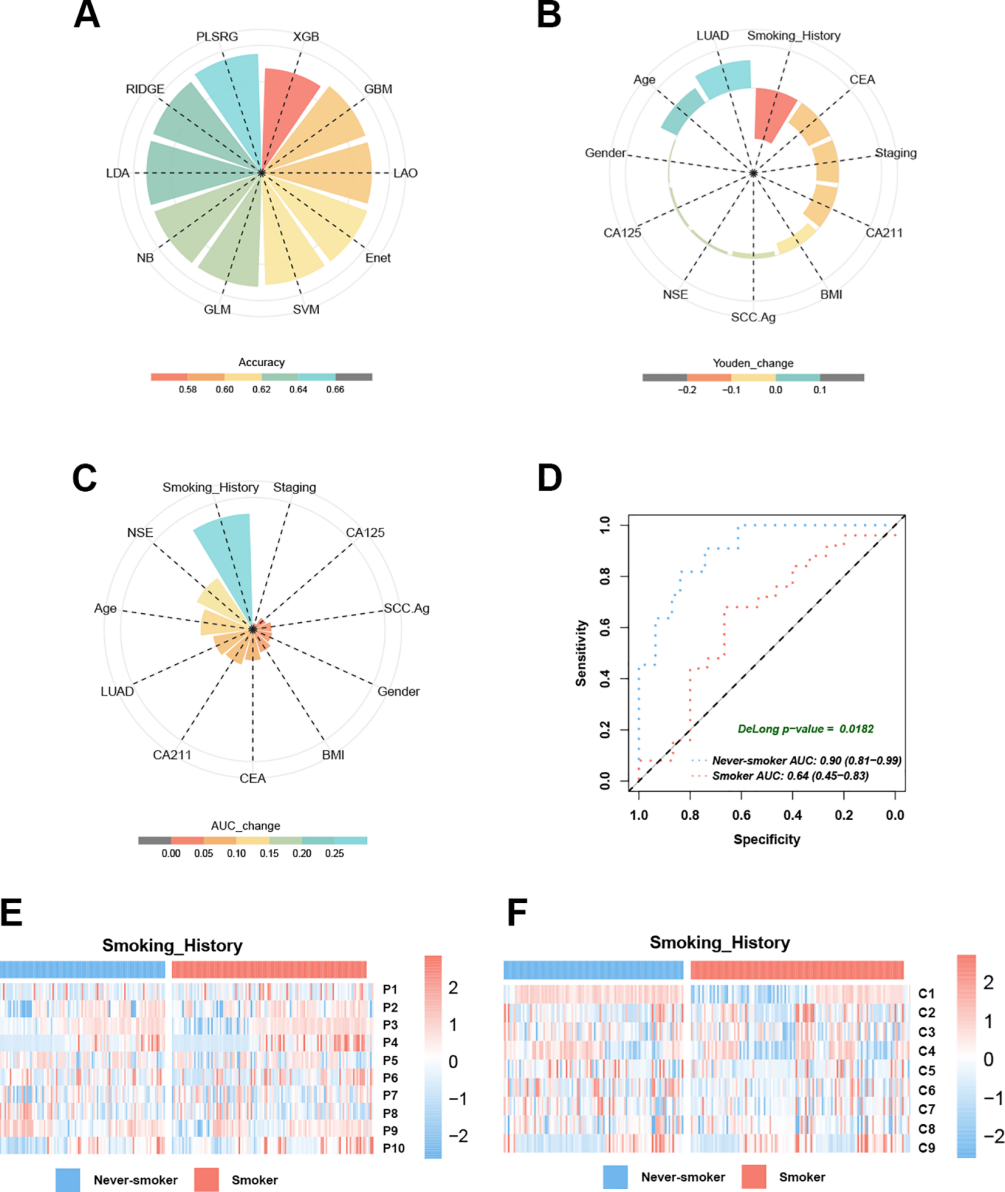
Smoking history emerges as a critical determinant influencing EGFR prediction model performance. **(A)** The pie chart demonstrates the accuracy of 10 machine learning techniques calculated by LOOCV. **(B)** Smoking history dominates the PCS model performance decline in Youden’s index. **(C)** Smoking history dominates the PCS model performance decline in the AUC. **(D)** The AUC curves of the PCS model for predicting EGFR mutation in smokers and never-smokers with NSCLC. **(E)** Heatmaps reveal the different distribution modules of PET features between smokers and never-smokers. **(F)** Heatmaps reveal the different distribution modules of CT features between smokers and never-smokers.

To assess prediction accuracy at the individual level, *EGFR* mutation probabilities for each patient were obtained using the PCS model across 10 machine learning algorithms via LOOCV. The plsRglm algorithm exhibited the highest concordance between predicted and observed EGFR status (**Figure 6A**).

The 11 baseline clinical characteristics were dichotomized using median-based cutoffs to identify factors associated with model misclassification. Systematic comparison of Youden’s index across subgroups indicated that smoking history had the largest impact (mean reduction = 0.18; **Figure 6B**). Predictive performance was significantly reduced in smokers, with seven of 10 models showing Youden’s index decreases ≥ 0.1 compared with never-smokers (**Supplementary Figure S2**). In the validation cohort, the PCS model achieved a lower AUC in smokers (AUC = 0.64, 95% CI: 0.45–0.83) than in never-smokers (AUC = 0.90, 95% CI: 0.81–0.99; ΔAUC = 0.26, **Figure 6C**; DeLong test, *p* < 0.05, **Figure 6D**). In contrast, other baseline characteristics—including age, gender, BMI, histological subtype, tumor marker levels, and clinical stage—had limited effects on the AUC of *EGFR* mutation prediction. Heatmap analysis revealed significant differences in PET and CT feature distributions between smokers and never-smokers (**Figures 6E, F**; **Table 5**), suggesting that smoking modulates PET/CT radiomic feature patterns. Collectively, these findings indicated that never-smokers represent the optimal population for radiomics-based EGFR mutation prediction in NSCLC.

**Table 5:** The distribution of PET and CT features in patients with/without smoking history.

Source	Abbreviation	Feature	P-value
PET	P1	original_firstorder_Kurtosis	0.055
PET	P2	original_glcm_Imc1	0.019
PET	P3	original_glszm_ZoneEntropy	0.035
PET	P4	wavelet-LLH_firstorder_Energy	0.000
PET	P5	wavelet-LLH_glcm_InverseVariance	0.821
PET	P6	wavelet-LHL_gldm_SmallDependenceEmphasis	0.020
PET	P7	wavelet-LHH_glcm_DifferenceAverage	0.556
PET	P8	wavelet-LHH_gldm_DependenceNonUniformityNormalized	0.303
PET	P9	wavelet-HLL_glcm_MCC	0.006
PET	P10	wavelet-HLH_glcm_ClusterShade	0.009
CT	C1	original_shape_Sphericity	0.001
CT	C2	original_ngtdm_Complexity	0.214
CT	C3	wavelet-LLH_glszm_SmallAreaEmphasis	0.925
CT	C4	wavelet-LHH_gldm_DependenceEntropy	0.045
CT	C5	wavelet-HLH_glcm_ClusterShade	0.632
CT	C6	wavelet-HHL_firstorder_Mean	0.278
CT	C7	wavelet-HHL_glcm_ClusterShade	0.458
CT	C8	wavelet-HHH_glcm_ClusterShade	0.295
CT	C9	wavelet-HHH_gldm_DependenceNonUniformityNormalized	0.030

## Discussion

In this study, we developed a PCS model based on ^18^F-FDG PET/CT radiomic features to predict EGFR mutation status in NSCLC, through a comprehensive evaluation of 10 machine learning algorithms. Key clinical factors influencing model misclassification were identified to define the optimal patient population for radiomics-based prediction. Furthermore, integrating clinical variables into a PCS-nomogram enhanced predictive accuracy and facilitated the clinical translation of imaging-to-molecular profiling approaches.

The LASSO regression coefficients indicated that *EGFR*-mutant tumors exhibited significantly elevated glucose metabolism, greater CT image heterogeneity, and higher sphericity compared with wild-type tumors, consistent with previous observations (11, 22–25). From a tumor biology perspective, these characteristics may stem from the ability of *EGFR* mutations to amplify downstream signaling cascades, particularly MEK/ERK and PI3K/AKT/mTOR pathways (26, 27). Activation of these pathways promotes tumor proliferation while increasing cellular glucose uptake (28, 29). In addition, EGFR-driven tumorigenesis has been associated with dysregulated angiogenesis, leading to regional necrosis due to insufficient oxygen perfusion, which manifests as heterogeneous CT patterns (30). Consistent with previous quantitative shape analysis (25), our findings revealed that *EGFR*-mutated lung carcinoma exhibited significantly higher sphericity indices than wild-type tumors, providing an additional morphological biomarker for predicting *EGFR* mutation status.

The 10 commonly used machine learning algorithms in current radiomics research (31–33) were systematically evaluated in our study, and plsRglm was identified as the optimal approach, forming the basis of the PCS model. In both training and validation cohorts, the dual-modality PET/CT model achieved significantly higher AUCs than the PET-only model (*p* < 0.05) and marginally higher AUCs than the CT-only models, consistent with a previous report (25). Similarly, a meta-analysis by Nguyen et al. reported comparable results, including 35 studies evaluating artificial intelligence-based radiomics models for predicting EGFR mutations in NSCLC (34). Notably, CT-based radiomics models alone demonstrated reasonably good predictive performance for EGFR mutation status (35, 36). Therefore, PET/CT-based models should be regarded as complementary tools that may provide additional value in patients who already undergo PET/CT for staging or follow-up, owing to the fact that PET/CT use remains limited by cost and accessibility.

We further demonstrated that combining the PCS with clinical characteristics improved the prediction of EGFR mutation status in lung cancer patients, particularly when incorporating gender and smoking history. Epidemiological studies have reported that *EGFR* mutations occur in 40%–50% of nonsmoking NSCLC patients compared to 10%–15% in smokers, confirming smoking status as an independent predictive factor for *EGFR* mutations (37, 38). Similarly, gender-associated differences have been documented, with Asian women with NSCLC exhibiting higher *EGFR* mutation rates (50%–60%) than men (20%–30%) (39, 40). Notably, the association between smoking history and *EGFR* mutations was stronger than that of gender (39, 40), consistent with the greater weight assigned to smoking in our PCS-nomogram scoring system.

Most importantly, we identified smoking status as the key determinant factor influencing the performance of imaging-based prediction models. Compared to other clinical characteristics, including histologic type, age, gender, and tumor biomarkers, smoking status had the greatest impact on model performance, as reflected by changes in the Youden’s index. Specifically, the predictive accuracy of imaging models was markedly lower in smokers than in never-smokers within the PCS model. This discrepancy was primarily attributable to the heterogeneity of imaging features between the two populations rather than differences in EGFR mutation prevalence (28.95% vs. 68.55%). Using a LOOCV strategy, each patient’s prediction was generated from a model trained on the remaining 276 samples, in which the EGFR mutation rate remained approximately 52.3%, consistent with that of the overall cohort. Mechanistically, several factors may explain why PET/CT-based EGFR mutation prediction models perform less effectively in smokers. First, previous studies have reported that smoking induces alternative mutations, including KRAS and TP53, through DNA damage mechanisms (41), which may produce distinct imaging features that obscure EGFR-related signatures. Second, smokers frequently present with comorbid pulmonary pathologies such as emphysema, chronic obstructive pulmonary disease, inflammation, and fibrosis (42), which can interfere with imaging biomarkers associated with *EGFR* mutations and further reduce model accuracy. Therefore, we recommend that future radiomics models for *EGFR* prediction consider restricting inclusion criteria based on smoking status to improve predictive validity.

Accurate integration of imaging and clinical features is critical in radiomics-based prediction studies. A recent review highlighted the strengths and limitations of different fusion strategies in this context (43). Early fusion, which combines imaging and clinical variables at the model training stage, can enhance predictive performance when sample size is sufficient and feature dimensions are balanced; however, in moderate-sized cohorts with a large imbalance between imaging and clinical features, it may lead to overfitting and obscure clinically meaningful predictors (44). In contrast, late fusion develops separate models for each modality and integrates their outputs at the decision level, offering greater interpretability and flexibility (45, 46). In the present study, given the moderate sample size and the disparity between imaging and clinical features, we adopted a more appropriate late fusion framework, which was confirmed to outperform early fusion strategy in multiple comparative studies under similar conditions (47–49). This approach also allowed independent analysis of clinical factors, enabling the identification of key contributors to model misclassification and the precise definition of the optimal patient population for machine learning-based EGFR mutation prediction.

Our study has several limitations. First, this was a single-center study, limiting sample diversity. External validation using multicenter cohorts is required to enhance model generalizability and robustness. The designation of smoking as a determinant factor of model performance also requires validation in larger cohorts. Second, preclinical studies have developed *EGFR*-targeted radiotracers, such as ^18^F-MPG, which may offer more specific mutation-related imaging features than conventional FDG PET/CT, providing greater precision for radiomics-based characterization of *EGFR* status (50, 51). Finally, the PCS-nomogram workflow involves multiple steps, including VOI delineation and feature extraction, necessitating integration into standardized clinical software to facilitate broader implementation.

In summary, our PCS-nomogram demonstrated the potential to noninvasively and accurately predict *EGFR* mutation status in NSCLC patients using baseline PET/CT imaging combined with clinical characteristics. This model could serve as a valuable complementary tool for patients with insufficient biopsy samples or contraindications to invasive procedures. Moreover, our findings revealed that radiomics-based prediction was more reliable in never-smokers, whereas smoking history acted as a major confounding factor contributing to prediction failure. These insights advance the clinical application of “imaging-to-molecular diagnosis”, guide patient selection, and hold promise for translation into clinical benefits.

## Data Availability

The original contributions presented in the study are included in the article/**Supplementary Material**. Further inquiries can be directed to the corresponding author.
