# Supplementary material for: Risk factors for misclassification in predicting EGFR mutation status using PET/CT imaging in non-small cell lung cancer patients
Source: Front Oncol. 2025 Dec 3;15:1702905. doi: 10.3389/fonc.2025.1702905 (PMC12708245; doi:10.3389/fonc.2025.1702905)

#### **Supplementary Material**

**Risk Factors for Misclassification in Predicting EGFR Mutation Status Using PET/CT Imaging Features in Non-Small Cell Lung Cancer Patients**

Jiali Li^1#^, Zihang Zeng^2#^, Jie Chen^1^, Tianxing Fang^1^, Hongjun Liu^1^, Yong He^1*^

^1^Department of Nuclear Medicine, Zhongnan Hospital of Wuhan University, Wuhan, China

^2^ Department of Radiation and Medical Oncology, Zhongnan Hospital of Wuhan University, Wuhan, China

**#contribute equally to this manuscript**

*** Correspondence:**Yong He
[heyong@whu.edu.cn](mailto:heyong@whu.edu.cn)

Table S1-S2: Page 2-3

Figure S1-S2: Page 4

**Supplementary Tables**

**Table S1: Diagnostic efficiency of different models with PET signatures in the training and validation cohorts.**

|  | Model_name | AUC | Accuracy | Sensitivity | Specificity | PPV | NPV |
| --- | --- | --- | --- | --- | --- | --- | --- |
| Training | XGB | 1 (1.00-1.00) | 1 (1.00-1.00) | 1 (0.96-1) | 1 (0.96-1) | 1 (0.98-1) | 1 (0.96-1) |
| Training | SVM | 0.82 (0.76-0.87) | 0.76 (0.7-0.82) | 0.73 (0.63-0.81) | 0.8 (0.71-0.87) | 0.84 (0.78-0.89) | 0.69 (0.61-0.76) |
| Training | RIDGE | 0.68 (0.61-0.76) | 0.64 (0.57-0.71) | 0.64 (0.54-0.73) | 0.65 (0.55-0.74) | 0.68 (0.6-0.75) | 0.6 (0.51-0.68) |
| Training | PLSRG | 0.65 (0.57-0.73) | 0.65 (0.58-0.72) | 0.64 (0.54-0.73) | 0.66 (0.56-0.75) | 0.69 (0.61-0.76) | 0.6 (0.52-0.68) |
| Training | NB | 0.64 (0.56-0.71) | 0.62 (0.55-0.69) | 0.66 (0.56-0.75) | 0.59 (0.49-0.68) | 0.5 (0.43-0.57) | 0.74 (0.66-0.81) |
| Training | LDA | 0.68 (0.61-0.76) | 0.64 (0.57-0.71) | 0.65 (0.55-0.74) | 0.63 (0.53-0.72) | 0.63 (0.55-0.7) | 0.65 (0.56-0.73) |
| Training | LAO | 0.69 (0.61-0.76) | 0.63 (0.56-0.7) | 0.66 (0.56-0.75) | 0.62 (0.52-0.71) | 0.58 (0.5-0.65) | 0.69 (0.6-0.76) |
| Training | GLM | 0.68 (0.61-0.76) | 0.63 (0.56-0.7) | 0.64 (0.54-0.73) | 0.63 (0.53-0.72) | 0.62 (0.54-0.69) | 0.65 (0.56-0.73) |
| Training | GBM | 0.86 (0.81-0.91) | 0.78 (0.71-0.83) | 0.73 (0.63-0.81) | 0.85 (0.77-0.91) | 0.89 (0.83-0.93) | 0.67 (0.59-0.74) |
| Training | Enet | 0.69 (0.61-0.76) | 0.64 (0.57-0.71) | 0.66 (0.56-0.75) | 0.63 (0.53-0.72) | 0.6 (0.52-0.67) | 0.68 (0.59-0.76) |
| Validation | NB | 0.7 (0.58-0.81) | 0.7 (0.59-0.79) | 0.73 (0.59-0.84) | 0.66 (0.5-0.79) | 0.74 (0.63-0.83) | 0.64 (0.5-0.76) |
| Validation | PLSRG | 0.68 (0.56-0.79) | 0.7 (0.59-0.79) | 0.7 (0.56-0.81) | 0.7 (0.54-0.82) | 0.83 (0.72-0.9) | 0.53 (0.39-0.66) |
| Validation | SVM | 0.61 (0.48-0.73) | 0.6 (0.49-0.71) | 0.67 (0.53-0.79) | 0.54 (0.38-0.69) | 0.6 (0.49-0.7) | 0.61 (0.47-0.74) |
| Validation | RIDGE | 0.61 (0.48-0.73) | 0.63 (0.51-0.73) | 0.65 (0.51-0.77) | 0.58 (0.42-0.73) | 0.72 (0.6-0.81) | 0.5 (0.37-0.63) |
| Validation | GBM | 0.59 (0.47-0.72) | 0.61 (0.5-0.72) | 0.74 (0.6-0.84) | 0.54 (0.38-0.69) | 0.49 (0.38-0.6) | 0.78 (0.63-0.88) |
| Validation | XGB | 0.58 (0.45-0.7) | 0.58 (0.46-0.69) | 0.73 (0.59-0.84) | 0.51 (0.35-0.66) | 0.4 (0.3-0.51) | 0.81 (0.66-0.9) |
| Validation | LDA | 0.54 (0.42-0.67) | 0.55 (0.44-0.66) | 0.63 (0.49-0.75) | 0.49 (0.34-0.65) | 0.51 (0.4-0.62) | 0.61 (0.47-0.73) |
| Validation | Enet | 0.54 (0.41-0.66) | 0.55 (0.44-0.66) | 0.65 (0.51-0.77) | 0.49 (0.34-0.65) | 0.47 (0.36-0.58) | 0.67 (0.53-0.79) |
| Validation | LAO | 0.53 (0.41-0.66) | 0.51 (0.39-0.62) | 0.8 (0.67-0.89) | 0.47 (0.32-0.63) | 0.17 (0.1-0.26) | 0.94 (0.81-0.98) |
| Validation | GLM | 0.53 (0.4-0.66) | 0.58 (0.46-0.69) | 0.6 (0.46-0.73) | 0.52 (0.36-0.67) | 0.77 (0.66-0.85) | 0.33 (0.22-0.47) |

AUC, area under the curve; CI, confidence interval; PPV, positive predictive value; NPV, negative predictive value

**Table S2: Diagnostic efficiency of different models with CT signatures in the training and validation cohorts.**

| Task | Model_name | AUC | Accuracy | Sensitivity | Specificity | PPV | NPV |
| --- | --- | --- | --- | --- | --- | --- | --- |
| Training | XGB | 1 (0.98-1.00) | 1 (0.98-1.00) | 1 (0.96-1) | 1 (0.96-1) | 1 (0.98-1) | 1 (0.96-1) |
| Training | SVM | 0.88 (0.83-0.93) | 0.81 (0.75-0.86) | 0.82 (0.73-0.88) | 0.8 (0.71-0.87) | 0.8 (0.74-0.85) | 0.82 (0.74-0.88) |
| Training | GBM | 0.85 (0.79-0.9) | 0.79 (0.73-0.85) | 0.8 (0.71-0.87) | 0.79 (0.7-0.86) | 0.79 (0.72-0.84) | 0.8 (0.72-0.86) |
| Training | RIDGE | 0.73 (0.66-0.8) | 0.69 (0.62-0.75) | 0.64 (0.54-0.73) | 0.81 (0.72-0.88) | 0.89 (0.83-0.93) | 0.48 (0.4-0.56) |
| Training | LDA | 0.73 (0.66-0.8) | 0.69 (0.62-0.75) | 0.65 (0.55-0.74) | 0.76 (0.67-0.83) | 0.84 (0.77-0.89) | 0.53 (0.45-0.61) |
| Training | LAO | 0.73 (0.66-0.8) | 0.69 (0.62-0.75) | 0.64 (0.54-0.73) | 0.81 (0.72-0.88) | 0.89 (0.83-0.93) | 0.49 (0.41-0.57) |
| Training | GLM | 0.73 (0.66-0.8) | 0.69 (0.62-0.75) | 0.64 (0.54-0.73) | 0.81 (0.72-0.88) | 0.89 (0.83-0.93) | 0.49 (0.41-0.57) |
| Training | Enet | 0.73 (0.66-0.8) | 0.69 (0.62-0.75) | 0.64 (0.54-0.73) | 0.81 (0.72-0.88) | 0.89 (0.83-0.93) | 0.49 (0.41-0.57) |
| Training | PLSRG | 0.71 (0.64-0.78) | 0.69 (0.62-0.75) | 0.7 (0.6-0.78) | 0.68 (0.58-0.76) | 0.67 (0.6-0.74) | 0.71 (0.62-0.78) |
| Training | NB | 0.7 (0.63-0.77) | 0.66 (0.59-0.73) | 0.66 (0.56-0.75) | 0.67 (0.57-0.76) | 0.7 (0.62-0.77) | 0.63 (0.54-0.71) |
| Validation | PLSRG | 0.73 (0.63-0.84) | 0.71 (0.6-0.81) | 0.77 (0.63-0.87) | 0.65 (0.49-0.78) | 0.7 (0.59-0.79) | 0.72 (0.57-0.83) |
| Validation | RIDGE | 0.72 (0.61-0.83) | 0.69 (0.58-0.78) | 0.73 (0.59-0.84) | 0.63 (0.47-0.77) | 0.7 (0.59-0.79) | 0.67 (0.52-0.79) |
| Validation | LDA | 0.72 (0.61-0.82) | 0.63 (0.51-0.73) | 0.9 (0.78-0.96) | 0.54 (0.38-0.69) | 0.38 (0.29-0.48) | 0.94 (0.79-0.99) |
| Validation | GLM | 0.72 (0.61-0.83) | 0.64 (0.53-0.74) | 0.9 (0.78-0.96) | 0.55 (0.39-0.7) | 0.4 (0.31-0.5) | 0.94 (0.79-0.99) |
| Validation | LAO | 0.71 (0.61-0.82) | 0.64 (0.53-0.74) | 0.9 (0.78-0.96) | 0.55 (0.39-0.7) | 0.4 (0.31-0.5) | 0.94 (0.79-0.99) |
| Validation | Enet | 0.71 (0.6-0.82) | 0.64 (0.53-0.74) | 0.9 (0.78-0.96) | 0.55 (0.39-0.7) | 0.4 (0.31-0.5) | 0.94 (0.79-0.99) |
| Validation | NB | 0.7 (0.59-0.81) | 0.65 (0.54-0.75) | 0.8 (0.67-0.89) | 0.57 (0.41-0.72) | 0.51 (0.4-0.62) | 0.83 (0.68-0.92) |
| Validation | GBM | 0.68 (0.57-0.8) | 0.66 (0.55-0.76) | 0.67 (0.53-0.79) | 0.65 (0.49-0.78) | 0.81 (0.7-0.89) | 0.47 (0.34-0.61) |
| Validation | XGB | 0.64 (0.52-0.76) | 0.6 (0.49-0.71) | 0.73 (0.59-0.84) | 0.53 (0.37-0.68) | 0.47 (0.36-0.58) | 0.78 (0.63-0.88) |
| Validation | SVM | 0.63 (0.51-0.75) | 0.64 (0.53-0.74) | 0.79 (0.65-0.88) | 0.56 (0.4-0.71) | 0.49 (0.38-0.6) | 0.83 (0.68-0.92) |

AUC, area under the curve; CI, confidence interval; PPV, positive predictive value; NPV, negative predictive value

**Supplementary Figures**

**Figure S1 The PET (A-B) and CT features (C-D) selection with LASSO.**


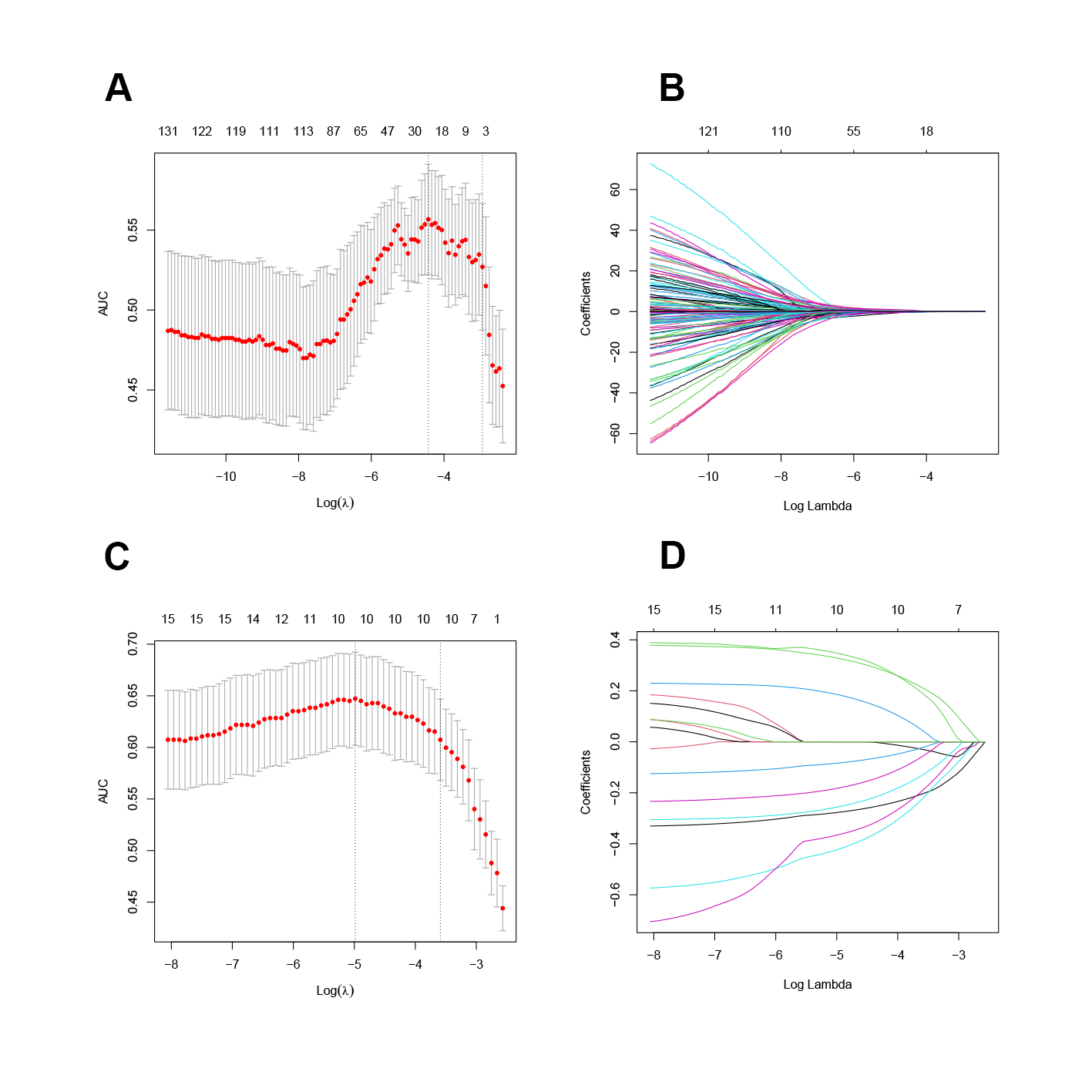


**Figure S2 :Smoking history altered the Youden index beyond 0.1 in the 7/10 models.**


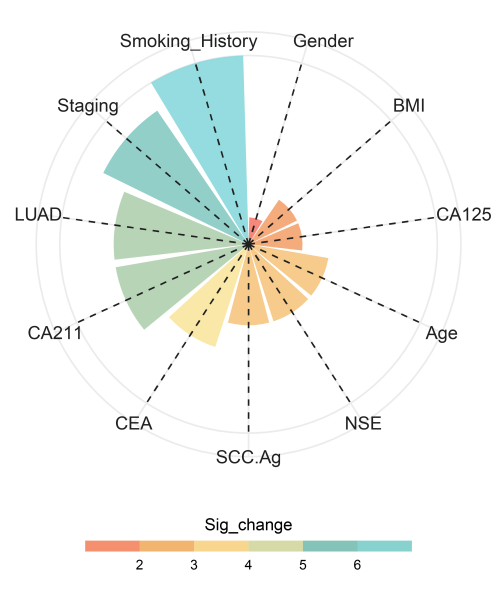

Supplement: Supplementary file 1 [file DataSheet1.docx]
